# Cervical cancer screening among HIV-positive women in urban Uganda: a cross sectional study

**DOI:** 10.1186/s12905-022-01743-9

**Published:** 2022-05-10

**Authors:** Najjuka Sarah Maria, Connie Olwit, Mark Mohan Kaggwa, Rose Chalo Nabirye, Tom Denis Ngabirano

**Affiliations:** 1 College of Health Sciences, Makerere University, Kampala, Uganda; 2 Department of Nursing, School of Health Sciences, College of Health Sciences Makerere University, Kampala, Uganda; 3 Department of Psychiatry, Faculty of Medicine, Mbarara University of Science and Technology, Mbarara, Uganda; 4 Faculty of Health Sciences, Busitema University, Busitema, Uganda

**Keywords:** Cervical cancer, Cervical cancer screening, HIV, Women, Health professionals, Uganda

## Abstract

**Background:**

Women living with Human Immunodeficiency Virus (HIV) are at a high risk for early development of cervical cancer. Adherence to cervical cancer prevention strategies in this population is vital for the early detection and treatment of cervical cancer. This study aimed to determine the prevalence and factors associated with cervical cancer screening among HIV-positive women attending an urban HIV care center in Uganda.

**Methods:**

This cross-sectional study included 205 HIV-positive women receiving care at an urban HIV care center. An interviewer-administered questionnaire was used to capture sociodemographic information, history of screening for cervical cancer, and reproductive health characteristics. Logistic regression analysis was used to determine the factors associated with cervical cancer screening.

**Results:**

Of the 205 HIV-positive women with a mean age of 37.5 ± 8.87 that participated in the study, majority (n = 201, 98%) were aware of cervical cancer screening. Ninety participants (44%) had ever been screened for cervical cancer and only 33 (16.1%) had been screened in the past year. Obtaining information about cancer of the cervix and cervical cancer screening from health care professionals was significantly associated with higher levels of cervical cancer screening (adjusted odds ratio = 5.61, 95% confidence interval: 2.50–12.61, *p *value < 0.001).

**Conclusion:**

This study highlights the low prevalence of cervical cancer screening among HIV-positive women and underscores the role of health professionals as an effective source of information on cervical cancer and cervical cancer screening. Patient education programs in HIV prevention and care facilities should emphasize cervical cancer screening messages to enhance the uptake of screening services.

## Introduction

Cancer is a term for diseases in which abnormal cells divide without control, invade nearby tissues and have the potential to spread to other parts of the body through blood and lymph systems [1]. Cancer of the cervix is a slow-growing cancer that forms in the cervical tissues and may involve the surrounding organs such as the vagina and uterus [2]. Globally, cancer of the cervix is the fourth most frequent cancer among women and was responsible for approximately 570,000 new cancer cases and 311,000 deaths in 2018 [3]. Cancer of the cervix is preventable through vaccination against Human Papillomavirus (HPV) and timely cervical cancer screening that aids early detection and treatment of pre-malignant lesions [4]. However, due to the lack of well-organized screening and HPV vaccination systems, almost 90% of cervical cancer morbidity and mortality occurs in low and middle-income countries (LMICs) [5, 6]. In these countries, cancer of the cervix is the second most common cancer and the leading cause of cancer-related deaths among women [7].

Uganda is among the top ten countries with the highest incidence of cervical cancer (28.8/100,000 per year) globally and ranks second in Eastern Africa with about 6413 new cases and 4301 deaths annually [8, 9]. In 2020, about 35.7% of new cancer cases among Ugandan women were due to cervical cancer and approximately 11 million Ugandan women are at risk of cervical cancer, caused by a sexually transmitted persistent infection with oncogenic sub-types of HPV; mainly HPV 16 and HPV 18 [8, 10, 11]. Risk factors for cervical cancer include multiple sexual partners, young age at first sexual intercourse, high parity, and prolonged use of oral contraceptives [12]. In addition, infection with Human Immunodeficiency Virus (HIV) is associated with a higher incidence of persistent HPV infection resulting in early development, rapid progression, malignant transformation of premalignant lesions and increased aggressiveness of already existing cancerous lesions [13, 14]. Unfortunately, HIV is one of the most common sexually transmitted diseases in the country with an overall prevalence rate of 6.2% and 7.6% among women aged 15–64 years according to the Uganda population-based HIV-impact assessment 2016–2017.

Due to the high susceptibility of HIV-positive women to cancer of the cervix, the Centers for Disease Control and prevention (CDC) and the Uganda national HIV treatment guidelines recommend annual screening in this population [7]. As such, Uganda developed a national strategic plan for cervical cancer prevention and control with the objective of disseminating information about cervical cancer prevention and treatment to 90% of its citizens including screening and treating 80% of eligible women [15]. However, no specific screening program has been designed for women living with HIV/AIDs to promote screening frequency in this high-risk population. In addition, there has been failure to integrate cervical cancer screening services in HIV care services due to limited resources. As a result, women living with HIV mainly access cervical cancer screening services at some but not all HIV-care facilities, sexual and reproductive health facilities and through occasional community screening outreaches. Never the less, women still face challenges while accessing screening services including long distances to the screening facilities, long waiting time at the facility, unaffordability of the services and some women rate the screening services as poor [16].

Despite implementation of the see-and-treat strategy (visualization of the cervix with acetic acid with diagnostic colposcopy, and treatment with cryotherapy), a simple and affordable approach to screening in Uganda [17], the prevalence of cervical cancer screening remains below average in both the general population and among women living with HIV [18–20]. There is limited literature regarding the screening practices of HIV-positive women in Uganda. The only existing national-wide study that explored the uptake and correlates of cervical cancer screening among HIV-infected women attending HIV care in Uganda found screening uptake at 30.3%, highest in Kampala- the capital city of Uganda (39.1%) [20]. Our study was conducted at an urban HIV care clinic in Kampala with improved access to screening services from neighboring health facilities. It aimed to re-evaluate the prevalence of cervical cancer screening among HIV-positive women in an urban setting and to explore the factors associated with increased screening in this population, to identify modifiable factors that could be targeted to enhance screening frequency among HIV-positive women national-wide.

## Materials and methods

### Study design, setting and population

This was a cross-sectional study among HIV-positive women attending the largest urban adult HIV care facility under The AIDS Support Organization (TASO) located at Mulago in Kampala, the capital of Uganda. TASO is one of the largest non-governmental organizations providing HIV/AIDS services in Africa and was founded in 1987 with a mission of contributing to the process of preventing HIV infection, restoring hope and improving the quality of life of people, families and communities affected by HIV infection and disease, with over 200,000 clients since its foundation. In Uganda, TASO operates in service sites distributed across the country including Mulago, Entebbe, Masaka, Mbale, Mbarara, Tororo, and Gulu. The TASO HIV care facility in Mulago is an outpatient clinic that primarily serves clients from the surrounding urban areas for instance, Mulago, Wandegeya, Bwaise, Kamwokya, Ntinda, and others. The clinic operated on three days per week with an average of 150 patients per day, about 70% of which were women.

### Sample size determination and sampling

We used the Kish Leslie formula, $n=\frac{{Z}^{2}p(1-p)}{{d}^{2}}$ to compute a sample size of 205 participants, where n—estimated sample size, Z—standard normal value corresponding to the level of confidence (1.96), p—estimated proportion of the study population with variable of interest (HIV-positive women who had ever screened for cervical cancer) and d—degree of precision of the study. This sample was based on a proportion of 15.9% reported in a study conducted in Malawi [21], due to lack of a prevalence study among Ugandan HIV-positive women at the time of the study design and we considered a 5% margin of error and 95% confidence interval. Participants were recruited in May 2017 by systematic random sampling. Considering the average number of female clients attending the clinic per day (105), a sampling frame (N = 945) was calculated based on the fact that the facility operated on three clinic days per week and that data was collected for a period of three weeks. A sampling interval (N/n) was then calculated to be 5. On each day of data collection, we randomly selected the first respondent and then selected every 5th eligible client who attended the clinic in their order of arrival to the facility.

### Eligibility criteria

HIV-positive women aged between 18 and 65 years were eligible to participate in the study. Clients that were too ill and those with history of total hysterectomy (n = 3) were excluded from participation in the study.

### Study instrument

Data were collected using an investigator-developed (based on extensive literature review) interviewer administered semi-structured questionnaire. This was pretested among 25 clients at the same facility, two weeks prior to data collection. The questionnaire was translated in Luganda (a language commonly used by patients attending the clinic), back-translated and administered in either Luganda or English. It captured respondents’ socio-demographic characteristics such as age, marital status, parity, level of education, and employment status; reproductive health characteristics such as age at first intercourse, contraceptive use, awareness of cervical cancer, awareness of cervical cancer screening, source of information about cervical cancer, knowledge of a cervical cancer screening facility, cost of cervical cancer screening, affordability of cervical cancer services, uptake of cervical cancer screening, previous screening practices, and how many times they had ever screened for cervical cancer.

### Ethical considerations

Following ethical approval by Makerere University College of Health Sciences School of Health Sciences Research and Ethics Committee (SHSREC REF 2017-020) and obtaining administrative clearance, the principal investigator approached participants through the out-patient clinic after they had utilized various services from the clinic. Eligible participants were taken to a private room, provided with information about the study, and those who voluntarily accepted to participate in the study were given a consent form to read prior to the interview. For participants who were illiterate, informed consent was obtained from a legally authorized representative and or legal guardian. Participants were given enough time to comprehend the information in the consent form and the principal investigator was available to answer any questions. Those who provided informed consent were then interviewed using a semi-structured questionnaire which was filled after clients had been attended by their HIV care providers.

### Statistical analysis

The collected data were entered into a computer using Statistical Package for Social Sciences (SPSS) version 21.0. It was cleaned and categorized into meaningful units and imported into STATA version 16.0 for statistical analysis. Frequencies and percentages were used to present categorical data, and means and standard deviations were used for the numerical data. Continuous variables that were not normally distributed were categorized into two categories based on their medians. Chi-square test was used to show statistical differences between the individuals who had screened for cervical cancer and those who had not screened. Logistic regression analysis was used to determine the factors associated with cervical cancer screening. Statistical significance was considered at a 95% confidence interval and a *p*-value less than 0.05.

## Results

### Socio-demographic and reproductive health characteristics

A total of 205 HIV-positive women with a mean age of 37.5 ± 8.87 years were included in the study. The majority, 57.1% (n = 117) of the participants were separated or widowed, and 67.3% (n = 138) had a parity of four and below. Only four participants had never heard of cervical cancer. A total of 201 (98%) women were aware of cervical cancer screening, and health care professionals were the dominant source of information for the participants 75.1% (n = 154). Less than half, 44.4% (n = 91), of the participants reported knowledge of a facility that offers cervical cancer screening, and 51 (56%), reported that the screening services were free. Details see Table 1.

**Table 1:** Relationship between participants’ characteristics and cervical cancer screening among HIV-positive women attending an Urban HIV care Clinic in Uganda (n = 205)

Variable (n = 205)	n (%)	Ever screening for cervical cancer		
		Yes n (%)	No n (%)	χ^2^ *P* value
Age in years (µ = 37.5 ± 8.87)				
≤ 36	105 (51.2)	37 (35.2)	68 (64.8)	6.56 (0.010)
> 36	100 (48.8)	53 (53.0)	47 (47.0)	
Marital status				
Single/never married	13 (6.3)	3 (23.1)	10 76.9)	2.44 (0.295)
Married/cohabiting	75 (36.6)	34 (45.3)	41 (54.7)	
Separated /widowed	117 (57.1)	53 (45.3)	64 (54.7)	
Parity (µ = 3.9 ± 2.2)				
≤ 4	138 (67.3)	56 (40.6)	82 (59.4)	0.90 (0.169)
> 4	33 (32.7)	34 (50.8)	33 (49.2)	
Level of education				
Uneducated/primary level	119 (58.1)	61(51.3)	58 (48.7)	6.37 (0.041)
Secondary level	63 (30.7)	22 (34.9)	41 (65.1)	
Tertiary level	23 (11.2)	07 (30.4)	16 (69.6)	
Employment status				
Employed	125 (41.0)	55 (44.0)	70 (56.0)	0.01(0.972)
Unemployed	80 (39.0)	35 (43.8)	45 (56.2)	
Age at first sexual intercourse (µ = 16.4 ± 2.5)				
≤ 16	115 (56.1)	58 (50.4)	57 (49.6)	4.54 (0.033)
> 16	90 (43.9)	32 (35.6)	58 (64.4)	
History of contraceptive use				
Yes	139 (67.8)	57 (41.0)	82 (59.0)	1.47 (0.225)
No	66 (32.2)	33 (43.9)	115 (56.1)	
Awareness of cervical cancer				
Yes	201 (98.0)	90 (44.8)	111 (52.2)	3.19 (0.074)
No	4 (2.0)	00 (0.0)	4 (100.0)	
Awareness of cervical cancer screening				
Yes	201 (98.0)	89 (44.3)	112 (55.7)	0.59 (0.442)
No	4 (2.0)	01 (25.0)	03 (75.0)	
Source of information (n = 201)				
Health professional ara>	154 (75.1)	81 (52.6)	73 9 (47.4	19.00 (< 0.001)
Media/friends/relatives	51 (24.9)	09 (17.7)	42 (82.3)	
Knowledge of a cervical cancer screening facility				
Yes	91 (44.4)	43 (47.3)	48 (52.8)	0.75 (0.390)
No	114 (55.6)	47 (41.2)	67 (58.8)	
Cost of cervical cancer screening service (n = 91)				
Free	51 (56.0)	27 (52.9)	24 (47.1)	1.50 (0.220)
Not free	40 (44.0)	16 (40.0)	24 (60.0)	
Affordability of the screening service (n = 40)				
Affordable	0	0 (0.0)	0 (0.0)	–
Not affordable	40 (100%)	16 (40.0)	24 (60.0)	

**Fig. 1:**
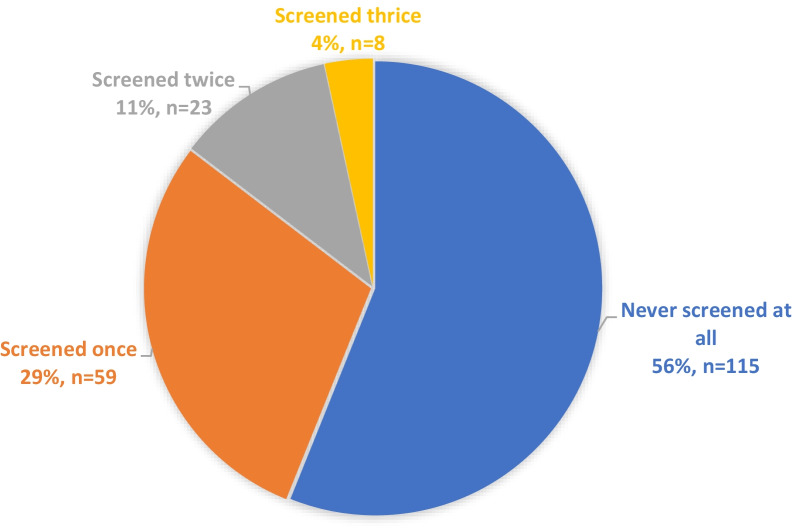
Life time cervical cancer screening frequency among HIV-positive women aged 18–65 years attending an Urban HIV care Clinic in Uganda (N = 205)

Less than half, 90 (44%), of the participants had ever screened for cancer of the cervix (Fig. 1) and the majority 83.9% (n = 172), had last screened more than a year ago.

There were statistical differences between individuals who had screened for cervical cancer and those who had not, among the following characteristics: age, level of education, age at first sexual intercourse, and source of information about cervical cancer. The highest difference was between individuals who had screened and received information from health care providers χ^2^ = 19.00, *p* ≤ 0.001. Approximately 53% of individuals aged above 36 years had screened for cervical cancer, which was higher than those 36 years and below, 35.2% (χ^2^ = 6.56, *p *value = 0.010). More women who had screened for cervical cancer, 51.3%, attained below the secondary level of education compared to 34.9% and 30.4% for women who had attained secondary and tertiary levels of education, respectively, (χ^2^ = 6.37, *p *value = 0.041). Participants who had hat their first sexual intercourse at 16 years or below had screened more for cervical cancer (50.4%) than for 35.6% of those who started sexual intercourse above 16 years (χ^2^ = 4.54, *p *value = 0.033) Table 1.

### Factors associated with cervical cancer screening

At bi-variable analysis, being above 36 years (crude odds ratio [cOR] = 2.07, 95% confidence interval [CI]: 1.18–3.63, *p *value = 0.011), being uneducated or having acquired primary level education (cOR = 2.07, CI 1.16–3.67, *p *value = 0.013), and health care professionals being the source of information about cervical cancer and screening (cOR = 5.12, CI: 2.36–11.37, *p *value ≤ 0.001) increased the likelihood of screening for cervical cancer. The likelihood of screening reduced if an individual had first sexual intercourse above 16 years (cOR = 0.54, CI 0.31–0.95, *p *value = 0.034) (Table 2). At multivariable analysis, only source of information was found to be a significant factor associated with cervical cancer screening. It increased the odds of screening for cervical cancer 5.61 times among those who received information from health workers compared to those who received information from other sources (Adjusted odds ratio = 5.61, CI 2.50–12.61, *p* ≤ 0.001). For details see Table 2.

**Table 2:** Logistic regression analysis for factors associated with cervical cancer screening among HIV-positive women in Uganda (n = 205)

Variable (n = 205)	Bivariate analyses		Multivariate analyses	
	Crude odds ratio (95% confidence interval)	*p *value	Adjusted odds ratio (95% confidence interval	*p *value
Age				
≤ 36	1.00		1.00	
> 36	2.07 (1.18–3.63)	0.011	1.73 (0.92–3.24)	0.086
Marital status				
Married	1.10 (0.62–1.94)	0.754	–	–
Not married	1.00		–	–
Parity				
≤ 4	1.00		–	–
> 4	1.51 (0.84–2.71)	0.170	–	–
Level of education				
Uneducated/primary	2.07 (1.16–3.67)	0.013	1.69 (0.86–3.30)	0.122
Secondary/tertiary	1.00		1.00	
Employment status				
Unemployed	1.00		–	–
Employed	1.01 (0.57–1.78)	0.972	–	–
Age at first sexual intercourse				
≤ 16	1.00		1.00	
> 16	0.54 (0.31–0.95)	0.034	0.70 (0.37–1.34)	0.288
History of contraceptive use				
Yes	0.70 (0.39–1.25)	0.226	–	–
No	1.00		–	–
Awareness of cervical cancer screening				
Yes	2.38 (0.24–23.31)	0.455	–	–
No	1.00		–	–
Source of information				
Health professional	5.12 (2.36–11.37)	< 0.001	5.61 (2.50–12.61)	< 0.001
Media/friends/relatives	1.00		1.00	
Knowledge of a cervical cancer screening facility				
Yes	1.28 (0.73–2.23)	0.388	–	–
No	1.00		–	–
Cost of cervical cancer screening service				
Free	1.69 (0.73–3.90)	0.22	–	–
Not free	1.00		–	–

## Discussion

Cervical cancer screening is recommended for high risk women especially those living with HIV to detect and treat precancerous lesions [22]. This study assessed the prevalence of cervical cancer screening among HIV-positive women and associated factors in an urban center in Uganda. We found the prevalence of cervical cancer screening among HIV-positive women at 44% which is lower than that reported in studies conducted in the USA (78%), [23], (83%), [24], Italy(91%), [25], and Canada (82%) [26]. This could be due to the better and well-structured health care systems and health care seeking behaviors of people in high income countries coupled with integrated cervical cancer screening and HIV care services that bring screening services close to HIV-positive women. These services have remained standalone programs in Uganda and other low and middle income countries (LMICs) which leads to missed opportunities for HIV-positive women to screen for cervical cancer despite frequent visits to HIV clinics for reviews and drug refills [27]. This is further complicated by poor health seeking behaviour of the Ugandan population [28]. The low prevalence of cervical cancer screening among women living with HIV has significant implications for the early detection and subsequent treatment of patients diagnosed with cancer of the cervix. Moreover, late diagnosis of cancer of the cervix is associated with poor treatment outcomes and is a strain to the already overstretched health care systems in LMICs such as Uganda. Unfortunately, screening behaviors among women living with HIV in LMICs have remained generally poor. For instance, studies in Ethiopia (10%) [29], Nigeria (9%) [30], and South Africa (32%) [31] reported lower proportions of cervical cancer screening than 44% in the present study. This variation could be explained by the increased awareness of cervical cancer and screening in the present study compared to that reported in above studies. In addition, this study was conducted in an urban setting where 60% of the participants were employed hence a higher social economic status which has been associated with a higher likelihood of screening [32]. Several studies affirm a higher prevalence of cervical cancer screening in urban areas than in rural areas [20, 33]. In this study however, cervical cancer screening practice was self-reported by participants yet, there are shreds of evidence that women tend to overestimate self-care practices when responding to questionnaires. Moreover, women with lower levels of education may be confused about what constitutes screening or whether having a gynecological examination means that one has taken a screening test. Therefore, the prevalence of cervical cancer screening among HIV-positive women may be lower than that reported in this and other studies.

In this study, obtaining information from health care professionals about cervical cancer and cervical cancer screening significantly increased cervical cancer screening by 5.6 times compared to other sources of information such as the media, friends or relatives. This is consistent with previous studies that highlight that health workers’ recommendation to screen for cervical cancer and access to health education in primary health facilities would enhance cervical cancer screening among women especially those living with HIV [19, 31, 34]. These findings suggest that access to primary care facilities could provide HIV-positive women with the opportunity of exposure to health professionals’ information and encouragement to utilize cervical cancer screening services. Moreover, HIV-positive women often visit health care facilities for routine care and drug refills, an opportunity that should be highly utilized. Health workers could also utilize other platforms such as the media and internet platforms to reach out to HIV-positive women through health education and empower women to seek cervical cancer screening services. Despite the fact that awareness of cervical cancer screening is crucial to enhance screening practice, improving awareness is not enough without access to care hence improving awareness should be part of a bigger strategy of offering regular screening through screening programs for HIV infected women.

Level of education was not a determinant of cervical cancer screening in this study. This was a surprising finding because education is a known enabling factor that helps women to have a better understanding of their risk of cervical cancer and influence their decisions to access health care services. Several studies have emphasized that education is an important predictor of the intention to screen and uptake of screening services [34, 35]. The variation in this study could be explained by the fact that women who were uneducated or who had attained only a primary level of education had screened more than those with higher levels of education. In addition, health education messages about cervical cancer could be designed in a simplified language that enhances comprehension by all HIV-positive women irrespective of their level of education. One study among Botswanan women also found no association between education and the prevalence of cervical cancer screening [36]. This finding suggests that interventions that aim at enhancing screening uptake should focus on all women regardless of their level of education.

Similarly, the cost of the screening service did not contribute significantly to the uptake of cervical cancer screening in this study. This finding could be due to the fact that more than half of the women who had knowledge of a screening facility in our study reported that services were free. Previous studies in LMICs also found that the ability to afford the screening service did not result in screening [30, 37]. This suggests that other factors such as cultural beliefs could influence the attitudes and perceptions of HIV positive women and may hinder women from utilizing the screening services. Various studies on the other hand highlight the cost of cervical cancer screening as a crucial barrier to screening especially when women perceive the cost to be high [38–40]. In this study, less than ten percent of the respondents had formal employment which could imply that the majority of HIV-positive women may not afford the screening services. It is also evident from our study that all participants who reported that screening services were not free also reported that they were not affordable. This finding suggests that HIV-positive women may find it difficult to pay for cervical cancer screening services resulting in low uptake of the screening services.

### Limitations

This study did not explore the possible reasons for low prevalence of cervical cancer screening in this high-risk population from the health workers’ perspective which calls for a qualitative study to investigate this phenomenon. Some important variables such as the number of years of HIV treatment were not captured which may have been important cofactors in determining individuals who screened for cervical cancer. Lastly, this was a cross sectional study and the actual causality for the low prevalence of cervical cancer screening cannot be made.

## Conclusion and recommendations

This study found a low prevalence of cervical cancer screening among HIV-positive women despite their high risk for the disease. Health professionals’ information and encouragement to women was a major determinant of the utilization of screening services. These findings add to previous studies to call for urgent integration of cervical cancer screening into HIV prevention and care services to prevent missed opportunities to screening. Patient education programs in HIV prevention and care facilities should include cervical cancer screening messages led by health professionals to enhance uptake of screening services.

## Data Availability

Data and materials will be available upon request from the corresponding author.
